# Lightweight Low-Rank Adaptation Vision Transformer Framework for Cervical Cancer Detection and Cervix Type Classification

**DOI:** 10.3390/bioengineering11050468

**Published:** 2024-05-08

**Authors:** Zhenchen Hong, Jingwei Xiong, Han Yang, Yu K. Mo

**Affiliations:** 1 Department of Physics and Astronomy, University of California, Riverside, CA 92521, USA; 2 Graduate Group in Biostatistics, University of California, Davis, CA 95616, USA; 3 Department of Chemistry, Columbia University, New York, NY 10027, USA; hy2565@columbia.edu; 4 Department of Computer Science, Indiana University, Bloomington, IN 47405, USA; moyu@iu.edu; 5 Department of Biology, Indiana University, Bloomington, IN 47405, USA

**Keywords:** cervical cancer, detection, vision transformer (ViT), low-rank adaptation (LoRA), cervix type classification, deep learning, computer-aided diagnosis

## Abstract

Cervical cancer is a major health concern worldwide, highlighting the urgent need for better early detection methods to improve outcomes for patients. In this study, we present a novel digital pathology classification approach that combines Low-Rank Adaptation (LoRA) with the Vision Transformer (ViT) model. This method is aimed at making cervix type classification more efficient through a deep learning classifier that does not require as much data. The key innovation is the use of LoRA, which allows for the effective training of the model with smaller datasets, making the most of the ability of ViT to represent visual information. This approach performs better than traditional Convolutional Neural Network (CNN) models, including Residual Networks (ResNets), especially when it comes to performance and the ability to generalize in situations where data are limited. Through thorough experiments and analysis on various dataset sizes, we found that our more streamlined classifier is highly accurate in spotting various cervical anomalies across several cases. This work advances the development of sophisticated computer-aided diagnostic systems, facilitating more rapid and accurate detection of cervical cancer, thereby significantly enhancing patient care outcomes.

## 1. Introduction

Cervical cancer is a significant public health concern, ranking as the fourth most prevalent cancer among women globally. It trails only breast, colorectal, and lung cancers in incidence, with over 500,000 new cases reported each year [1,2,3,4,5,6,7]. Even more alarming are the stark geographical disparities that exist in the global burden of cervical cancer, which reflect significant inequalities in access to preventive measures and healthcare services, reflecting the availability, coverage, and quality of preventive strategies, as well as the prevalence of risk factors. Approximately 90% of cervical cancer deaths occur in low- and middle-income countries (LMICs), underscoring the pressing need for improved access to effective diagnostic and treatment options in these regions [2,3]. In developing countries, women often face numerous barriers to accessing adequate cervical cancer screening programs. These obstacles include the high costs associated with regular examinations, limited awareness about the importance of screening, and insufficient access to medical facilities. As a result, individual patients in these regions are at a considerably higher risk of developing cervical cancer compared to those in more developed nations [8]. Implementing effective screening strategies can significantly reduce deaths caused by cervical cancer. Effective cervical cancer screening strategies have demonstrated a remarkable impact on reducing the lifetime risk of developing the disease. Studies have shown that these interventions can decrease the risk by a substantial 25% to 36%. Moreover, cervical cancer screening proves to be highly cost-effective, with estimates suggesting that the cost per year of life saved is less than $500. This highlights the significant public health benefits and economic value of implementing comprehensive cervical cancer screening programs [9]. As modern medical and computer technologies rapidly advance, numerous screening and diagnostic approaches now rely on computer-aided detection (CAD) architectures [10]. The importance of early detection and surgical intervention in the treatment of cervical cancer cannot be overstated, given that the disease often presents no symptoms in its initial stages. There are several methodologies employed for the early detection of cervical cancer, each with its own set of advantages. For example, colposcopy offers a direct visual inspection of the cervix through a specialized magnifying device, allowing for the identification of visible abnormalities or lesions that may suggest the presence of cervical cancer [11,12,13]; Also, the Papanicolaou (Pap) test or biopsy alongside human papillomavirus infection (HPV) typing tests, plays a crucial role in early diagnosis by examining cervical cells under a microscope to detect precancerous or cancerous modifications [14,15,16,17]. Moreover, biomarker testing on tumor samples yields insights into the tumor’s specific characteristics, aiding in the tailoring of treatment strategies. The advent of sequencing techniques over the past decade, particularly in analyzing HPV genotypes and cervical cancer [18,19,20,21,22], has marked a significant advance. These techniques enable the identification of genetic alterations linked to cervical cancer by detecting the virus and a wide array of HPV genotypes in clinically challenging samples, and then the genotype or evolutionary analysis [23,24,25,26,27] enhances our understanding of the disease’s genetic landscape, offering deeper insights into its mechanisms.

While the mentioned diagnostic modalities offer the potential for early-stage cervical cancer detection—thereby improving treatment efficacy, patient outcomes, and survival rates—certain methodologies present limitations in terms of cost-effectiveness and efficiency [9,28,29,30]. Specifically, the procurement of sequencing data for each patient needs substantial financial investment, and the evolutionary analysis of cancer samples demands significant time [31,32,33,34,35]. In contrast, cervix image screening via colposcopy emerges as a more cost-efficient and time-effective strategy [36,37,38], enabling the identification of precancerous changes or early-stage cancerous developments. Nevertheless, the early detection of cervical cancer remains a complex challenge. This complexity is compounded by instances of low-quality cervix image screening samples and the presence of subtle abnormalities that are challenging to discern, particularly during the disease’s initial stages. For example, the quality of cervix screening images varies due to discrepancies in collection methodologies, sample composition, and processing techniques, presenting a challenge in obtaining uniformly high-quality diagnostic images. And the morphological changes indicative of early-stage cervical cancer in cervix images are often subtle and difficult to differentiate from normal images, complicating the task of early detection. Also, the subjective evaluation of cervix image screening outcomes by pathologists can introduce variability in the identification of cervical abnormalities, underscoring the need for standardized interpretation frameworks. Lastly, while screening tests are designed to be sensitive, the occurrence of false-negative results is not uncommon, potentially delaying cervical cancer diagnosis and adversely affecting patient outcomes.

Despite the availability of extensive qualified datasets, the evaluation of cervix types—Type 1 Cervical Intraepithelial Neoplasia (CIN), Type 2 Squamous Intraepithelial Lesion (SIL), and Type 3 Dysplasia—from cervix screening images remains a complex and time-intensive task that necessitates the acumen of experienced clinicians. The difficulty in discerning between these cervix types from screening images is notably exacerbated by the intrinsic limitations of such images and the complex nature of the morphological changes within cervix tissue structures. In some recent studies, the results of colposcopy examinations are not consistently reproducible or precise. For one instance, false-negative rates vary widely, ranging from 13% to 69%, due to discrepancies in physician expertise and the region of the sample being examined [39]. And other studies also reported false-negative rates ranging from 25% to 57% specifically for biopsy samples identified as positive during colposcopy examinations [40]. Nonetheless, recent innovations in imaging technologies, including high-resolution microscopy and advanced digital imaging systems, have significantly improved the quality and definition of cervix screening images, thus aiding in the identification of nuanced abnormalities [41,42,43,44,45]. Furthermore, the progress in digital pathology platforms has enabled the digitization of histological slides, which supports remote access, facilitates image analysis, and promotes computer-aided diagnosis, thereby enhancing operational efficiency and encouraging collaboration among medical professionals. From an analytical standpoint, machine learning and deep learning models, trained on comprehensive datasets of cervix screening images, show promise in autonomously detecting and categorizing abnormal cervix types. The efficacious deployment of these sophisticated computer vision models underscores their wider applicability in biomedical research, heralding a new era of automated and precise analysis across various experimental paradigms [18,46,47,48,49,50,51]. These algorithms excel in identifying complex patterns and features imperceptible to the human eye, thereby improving the precision and reliability of cervical cancer screening efforts. Such technological advancements in artificial intelligence have markedly augmented the capabilities in disease detection, with computer-aided and AI-based methodologies revolutionizing the diagnostic processes for cervical cancer.

However, training advanced deep learning models on medical data, which is often constrained in volume, poses substantial challenges. These models, with millions of parameters, demand a considerable dataset to avert over-fitting and guarantee effective generalization. Nevertheless, the inherently limited scale of medical datasets—stemming from privacy issues, data collection hurdles, and ethical restrictions—renders the direct application of complex CNN frameworks on such data frequently untenable, potentially resulting in sub-optimal outcomes. To address the challenges of cervix type classification from cervix screening images using advanced CNN models, this study introduces a pioneering approach in digital pathology classification. It incorporates Low-Rank Adaptation (LoRA) into the Vision Transformer (ViT) architecture, designed to enhance the precision of cervix type categorization. Our methodology leverages LoRA to enable efficient model training on constrained datasets, thus exploiting the sophisticated visual representation prowess of Vision Transformers. In comparison with conventional models from the recent decade, such as VGG, GoogLeNet, ResNet, DenseNet and ResNeXt [4,52,53,54,55,56,57,58,59,60,61,62], our strategy achieves enhanced performance and exhibits remarkable generalization capabilities, especially in scenarios marked by limited data. Extensive experimentation and analytical scrutiny on benchmark datasets validate the efficacy of our integrated ViT with LoRA model in accurately identifying cervical cancer markers. This study marks a significant leap forward in advancing computer-aided diagnosis systems, paving new paths for the early detection and management of cervical cancer.

## 2. Review of Study

The availability of larger and more diverse datasets containing cervix images from varied populations and stages of disease has notably improved the performance of deep learning-based classifiers, simultaneously mitigating the risk of model over-fitting to specific traits of the training data. Nevertheless, the majority of existing literature emphasizes the accuracy and various other performance metrics on training sets, with scant attention paid to disclosing model performance on validation or test datasets as detailed in Table A1. Moreover, research involving patient biomedical images often encounters constraints in revealing actual data and trained models, thereby limiting opportunities for external validation of the models and data described in these studies [63,64,65]. Upon reviewing publicly available datasets, it is common to find that while training sets display high accuracy and other metrics, the performance on validation and test sets typically yields satisfactory yet variable outcomes.

The domain of cervical cancer detection has witnessed remarkable advancements through the adoption of deep learning and computer vision, offering a range of innovative strategies to improve diagnostic accuracy. Early research efforts [66] employed machine learning algorithms, notably K-NN, to distinguish between normal and pathological cervical tissues, yielding promising results in sensitivity and specificity. Subsequent studies [67] explored the utility of deep learning further in enhancing diagnostics for cervical cancer, including the development of automatic segmentation techniques for the cervical region and evaluating the performance of deep learning approaches against traditional methods, such as Pap smears.

A pivotal development in this field is the creation of the “Colposcopy Ensemble Network” (CYENET) [68], which applies a deep learning framework for the classification of colposcopy images into distinct categories to aid in cervical cancer detection. Trained on an extensive screening dataset, CYENET has exceeded the accuracy of established models like VGG16 and VGG19. Additionally, the ongoing investigation into deep convolutional neural networks (DCNNs) with a variety of optimizers signals a persistent effort to refine the accuracy in differentiating between benign and cancerous cervical images. The emergence of computer-aided diagnosis (CAD) systems, such as “CerCan·Net” [69], and novel approaches to image size optimization [70], mark significant strides towards leveraging deep learning in cervical cancer screenings. These advancements not only underscore the vast potential of deep learning in medical imaging but also pave the way for future research focused on optimizing neural networks for enhanced diagnostic accuracy and integrating machine learning innovations to improve outcomes in patient care.

## 3. Materials and Methods

### 3.1. Images Acquisition

MobileODT has implemented a Quality Assurance workflow to support remote supervision, enhancing the decision-making process for healthcare providers in rural areas. Improving this workflow to facilitate real-time assessments of patient treatment eligibility based on cervix type would significantly contribute to the early detection of cervical cancer. In a collaborative effort, MobileODT and Intel launched a classification contest on Kaggle [71]. This competition invites participants to develop an algorithm capable of accurately determining a woman’s cervix type from images, aiming to minimize ineffective treatments and ensure that patients receive the correct referrals for more specialized care if necessary.

**Figure 1:**
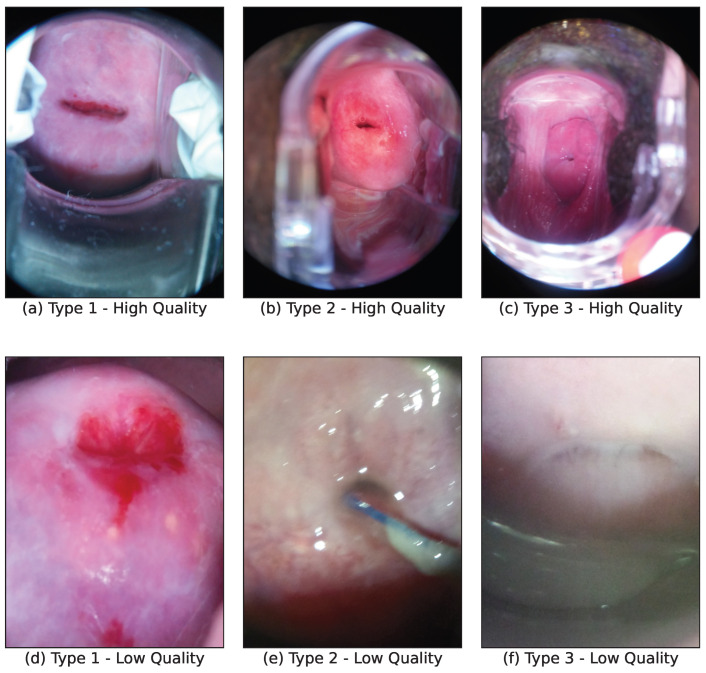
Sample dataset image. (**a**–**c**) are sample images of different cervix types with high quality; (**d**–**f**) are sample images of different cervix types with lower quality.

In a study involving 218,847 women in the older age group and 445,382 in the younger age group, researchers discovered a low incidence rate of cervical cancer during screening for Type 1 cervical intraepithelial neoplasia [72]. However, regular follow-up and monitoring are still crucial for women diagnosed with Type 1, as these lesions can potentially progress to higher grades of Type 1, which have a greater risk of developing into cervical cancer. Individuals with Type 2 and Type 3 cervixes require more extensive screening procedures. Detailed information about the distribution of cervix screening images in this dataset is provided in Table 1, and examples of these images are illustrated in Figure 1.

### 3.2. Cervix Type Classification Benchmarking Models

#### 3.2.1. AlexNet [73]

Introduced in 2012, AlexNet [73] marked a pivotal moment in the field of computer vision, scoring the top spot in the ImageNet dataset. This architecture, featuring eight layers including convolutional, max-pooling, and fully connected layers, incorporates novel elements such as rectified linear units (ReLUs), dropout regularization, and GPU acceleration. These innovations not only cut down training time but also set a new benchmark for subsequent neural network models, catalyzing a wave of advancements in deep learning.

#### 3.2.2. GoogLeNet [74]

GoogLeNet [74], also known as Inception-v1, introduced the inception module to the CNN landscape. This module supports the parallel use of various convolutional filter sizes within the same layer, balancing performance with computational efficiency. With 22 layers, including inception modules and a global average pooling strategy, GoogLeNet introduced auxiliary classifiers to mitigate the vanishing gradient problem, demonstrating high accuracy in image classification tasks and influencing future CNN designs.

#### 3.2.3. VGG (Visual Geometry Group) [75]

The VGG model [75], developed by the University of Oxford’s Visual Geometry Group in 2014, is celebrated for its straightforward yet effective architecture. With its series of convolutional layers followed by max-pooling, VGG exemplifies how deep and uniform structures can capture complex hierarchical features, thereby achieving remarkable accuracy in image classification challenges.

#### 3.2.4. ResNet [76]

ResNet [76], introduced in 2015, marked a turning point in deep learning by innovatively addressing the vanishing gradient problem with residual learning. It incorporates skip connections that directly add inputs to outputs, allowing for the seamless training of networks that are significantly deeper than previously possible. This design enables the network to learn residual functions with ease, ensuring that deeper network layers can learn identity functions as a default, thereby preventing the degradation problem. The widespread adoption of ResNet across various computer vision applications can be attributed to its remarkable efficiency in learning hierarchical features, facilitating advancements in deep neural network architectures and making it a foundational model in the field.

#### 3.2.5. DenseNet [77]

DenseNet [77], presented in 2017, offered a solution to the vanishing gradient problem by promoting feature reuse through its dense connectivity pattern, wherein each layer is connected to every other layer in a feed-forward fashion. With dense blocks and transition layers that manage parameter size, DenseNet achieves superior performance on image classification tasks, optimizing efficiency.

#### 3.2.6. ResNeXt [78]

Building on the successes of ResNet, ResNeXt [78] was introduced in 2017, presenting a novel way to increase the model’s capacity and performance without a substantial increase in complexity. The key innovation of ResNeXt lies in its use of “cardinality”, a dimension that represents the number of independent paths within the network. This concept allows ResNeXt to capture a wide array of features by aggregating transformations from multiple paths, effectively increasing the network’s robustness and efficiency without the need for a proportional rise in the parameters or computational demand.

### 3.3. A Cervix Type Classification Pipeline

**Figure 2:**
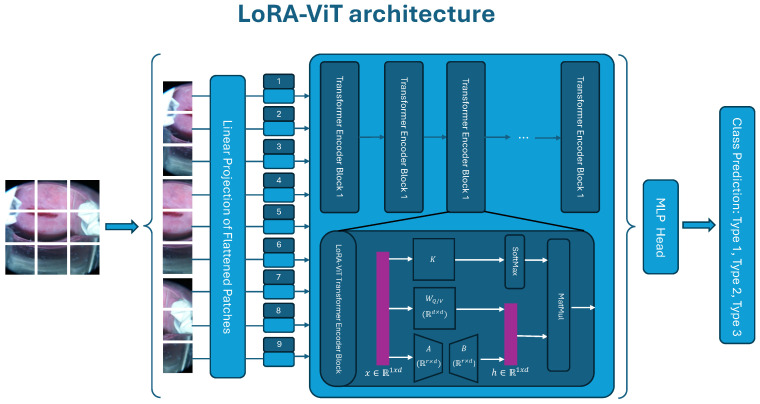
Schematic of the LoRA-ViT architecture for cervical cancer classification. The process begins by segmenting an image into fixed-size patches, linearly embedding each patch, adding positional embeddings, and then inputting the vectors into a standard Transformer encoder. The Transformer encoder is modified by incorporating low-rank decomposition matrices (denoted as A and B), which are injected into the fixed pre-trained query ($W_{Q}$) and value ($W_{V}$) projection matrices of each self-attention layer.

The system flow diagram showcasing the proposed method for cervix type is illustrated in Figure 2: The architecture commences by dividing an input cervical image into patches of fixed size. Each patch undergoes linear embedding, with positional embeddings added to retain spatial information. These embedded vectors are then inputted into a standard Transformer encoder, modified to integrate Low-Rank Adaptation (LoRA). Within each self-attention layer of the Transformer encoder, low-rank decomposition matrices (referred to as A and B) are introduced into the fixed pre-trained query ($W_{Q}$) and value ($W_{V}$) projection matrices. This facilitates efficient adaptation of the pre-trained Vision Transformer to the specific task of cervical cancer classification, while preserving the majority of the model’s parameters. The LoRA-ViT architecture facilitates the effective learning of task-specific representations with minimal computational overhead and mitigates the risk of over-fitting.

### 3.4. Low-Rank Adaptation (LoRA) and Vision Transformer (ViT)

Vision Transformer (ViT) [79] is a novel deep learning architecture that adapts the Transformer model, initially designed for natural language processing, to tasks in computer vision, notably image classification. ViT introduces a groundbreaking method by treating images as sequences of patches, mirroring the tokenization of words in language processing. This innovative approach revolutionizes image processing, leveraging the Transformer’s strengths in capturing complex relationships within sequences for improved vision tasks.

While ViT models stand out for their remarkable accuracy and enhanced generalizability across various tasks [80], their application to cervical cancer classification is fraught with challenges, especially within clinical settings. This is primarily due to the fact that ViT models, with their transformer architecture, are significantly larger in terms of parameter count compared to previous CNN-based models. This architecture demands a much larger dataset and longer training times for full parameter training compared to CNNs. When it comes to the task of classifying cervical cancer images, the available datasets are exceptionally limited (fewer than 800 images per class) compared to the vast ImageNet dataset [81] typically used to train ViTs, potentially leading to training failures under full parameter training conditions. Even if a sufficient number of images are available for training, the clinical imperative for efficient storage space utilization, minimal GPU resource use, and rapid processing of medical images adds another layer of complexity to the deployment of ViT models in this context.

Given the challenges of the extensive parameter size and the high data and training requirements when applying ViT models to cervical cancer classification, there emerges a compelling need for innovative training methods. Low-Rank Adaptation (LoRA) [82] by Microsoft provides an ingenious solution to this dilemma by adapting pre-trained vision models for use in robust cervical cancer detection systems without the need for complete fine-tuning. This method involves locking the weights of the pre-trained model and integrating trainable rank decomposition matrices into each layer of the Transformer architecture. By doing so, LoRA dramatically reduces the number of trainable parameters during the fine-tuning process. This reduction not only makes the training process more feasible with the datasets typical of medical imaging but also ensures efficient use of storage and GPU resources, aligning with the critical clinical requirements for space and speed [83].

In this study, we focus on the fact that the LoRA approach to training with limited data does not compromise on performance; it maintains or even enhances the model’s effectiveness compared to full-parameter training. In scenarios where extensive data are not available, which is often the case in medical contexts, the LoRA methodology is particularly advantageous. It provides a path to leverage the superior capabilities of ViT models over traditional CNNs for medical image classification tasks, such as cervical cancer detection, without the extensive resource commitments typically associated with these models. LoRA thus stands out not just for its efficiency and reduced computational demands but for maintaining high model quality, achieving a balance that is critically needed in the medical imaging domain.

As for implementation of the method architecture, we incorporate LoRA weights into each self-attention layer of a pre-trained ViT. During fine-tuning, the pre-trained query $W_{Q}$ and value projection matrices $W_{V}$ in a self-attention layer have their updates restricted by the introduced LoRA weights. These weights represent them with a low-rank decomposition and are expressed as the following:

(1) $$\begin{array}{c} h=W_{0}x+\Delta Wx=W_{0}x+BAx \end{array}$$

where $x\in R^{1\times d}$ is the input, and $h\in R^{1\times d}$ is the output features. Two low-rank matrices, $B\in R^{d\times r}$ and $A\in R^{r\times d}$, compose the weight change ΔW. At the onset of training, we employ a random Gaussian initialization for matrix *A* and initialize matrix *B* with zeros. Consequently, the product of matrices *B* and *A*, denoted as ΔW, is zero initially. The ranks *r* of these low-rank matrices are much smaller than the model dimension *d*, and we empirically set r=8 in our experiments. Usually, *r* should not be larger than 8, because the low-rank matrix amplification ability will be impaired when the rank is 64 in the experiment.

With this proposed model and pipeline architecture, our LoRA-based ViT classifier provides a solution by offering more accurate predictions of cervix types within a significantly shorter training period compared to the original ViT and other popular deep learning neural network models.

### 3.5. Performance Methods

The performance of the classification models was assessed using several objective evaluation metrics: accuracy, precision, recall, F1 score and Matthew Correlation Coefficient (MCC). These metrics rely on the true-positive (TP), true-negative (TN), false-negative (FN), and false-positive (FP) values of the models’ predictions for each cervix type included in the confusion matrix:

(2) $$Accuracy=\frac{TP+TN}{TP+TN+FP+FN}$$

(3) $$Precision=\frac{TP}{TP+FP}$$

(4) $$Recall=\frac{TP}{TP+FN}$$

(5) $$F1\;score=\frac{2*Precision*Recall}{Precision+Recall}$$

(6) $$MCC=\frac{TP*TN-FP*FN}{\sqrt{\left(TP+FP)*(TP+FN)*(TN+FP)*(TN+FN\right)}}$$

## 4. Results

### 4.1. Overall and Trainable Parameters

The landscape of deep learning models is marked by a rich diversity of architectures, each uniquely designed to address specific challenges in computer vision. State-of-the-art models such as ResNet, DenseNet, VGG, and GoogLeNet have gained widespread acclaim for their innovative methodologies and advanced architectures, establishing them as leading choices for computer-aided diagnosis applications of cervical cancer classification in recent years, details seen in the session of “Review of study”. These models have revolutionized the field of deep learning by introducing novel concepts and techniques that address critical challenges in medical image analysis. Despite the advancements these architectures offer, they share a common challenge: the increasing number of trainable parameters as models grow in complexity. This surge in parameters escalates the computational demands, affecting both training and inference phases. Therefore, finding a balance between model complexity and performance becomes paramount. Adopting optimization strategies such as parameter sharing, model pruning, and low-rank factorization is essential to manage these computational demands efficiently. For detailed insights into the dimensions of neural networks, including batch sizes and trainable parameters, Table 2 provides a comprehensive overview. The bold values are the best performance in the comparison across this paper.

Our method distinguishes itself by having the lowest count of trainable parameters among the leading deep learning architectures, with only 0.15 million trainable parameters. This figure is notably less than the 1% of parameters in the least complex model mentioned in our comparison. This dramatic reduction in trainable parameters enhances the efficiency of the training process and computational resource utilization across a wide array of datasets. Furthermore, when integrating Low-Rank Adaptation (LoRA) with the Vision Transformer (ViT), the model requires less than 0.2% of the trainable parameters found in the original ViT-base and merely 0.05% in ViT-huge. Although ViT demonstrates superior performance over all other models discussed, its substantial computational demands make the training process less feasible, particularly with limited datasets. Therefore, our ViT classifier, augmented with LoRA, presents a viable solution by enabling more precise cervix type predictions in significantly reduced training times compared to both the original ViT and other prevalent deep learning neural network models. By incorporating an optimized data loader and object-oriented programming-based image manipulation pipeline, our model achieves faster convergence rates with fewer trainable parameters and optimized architecture in Table A2, demonstrating the effectiveness of these optimizations in accelerating the model’s convergence. The systematic approach to image manipulation, coupled with the modular design of the object-oriented-based processing pipeline, enables seamless integration with our model architecture. This facilitates efficient experimentation with various pre-processing techniques and parameter configurations, empowering us to fine-tune the data processing pipeline for optimal model performance. Furthermore, the refactored data loader results in a more structured and manageable code base, contributing to the overall efficiency of the data-processing tasks.

### 4.2. Confusion Matrix of the Prediction Results

**Figure 3:**
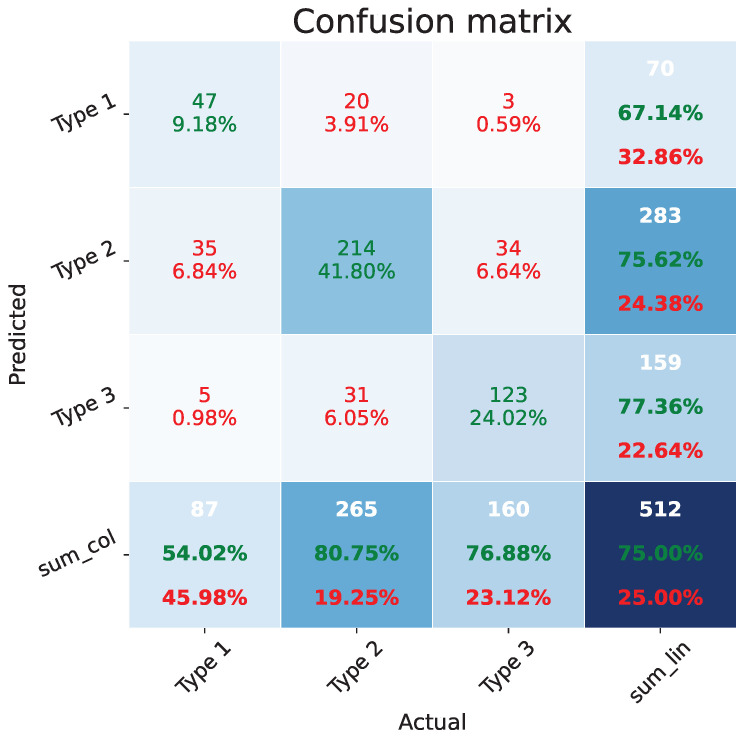
Confusion matrix. There are three different cervix types (Type 1, Type 2 and Type 3) in the matrix. (1) The 3 by 3 matrix at the top left corner is the confusion matrix of our proposed classifier. The first row is the counts of images for each category and the second row is the corresponding count percentage. The red values in the confusion matrix are the incorrect predictions, while the green ones are the correct predictions. (2) The last row and last columns and the aggregated statistics on the sample number and corresponding proportion. The white values are the sum counts of the predicted labels or actual labels on each row or column. The green values are the percentage of correct predictions, while the red ones are the percentage of incorrect predictions.

A confusion matrix for classification with three classes organizes model predictions into a grid with rows representing the actual classes and columns representing the predicted classes. Each cell in the matrix shows the count (or proportion) of instances for a specific combination of actual and predicted classes. As in Figure 3, our method performs well in the classification task of three cervix types with limited data. More than 75% of images are classified correctly as the corresponding cervix type (54.0% Type 1 accuracy, 80.6% Type 2 accuracy, and 76.9% Type 3 accuracy), which is much better than the other classifiers on the same dataset [84] with only 37.9% overall accuracy (33.0% Type 1 accuracy, 47.8% Type 2 accuracy, and 35.9% Type 3 accuracy).

### 4.3. Preliminary Results, Accuracy and Loss

**Figure 4:**
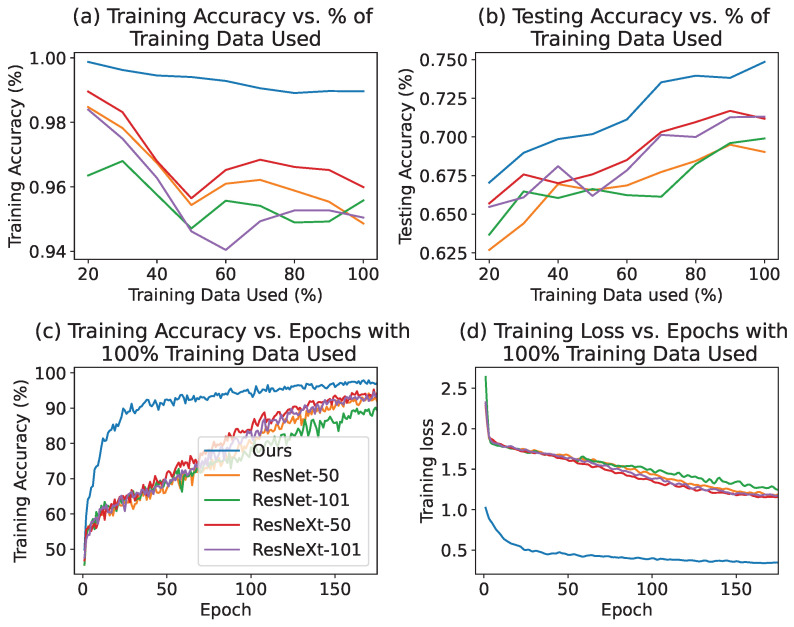
Preliminary results. (**a**) The Training Accuracy vs. Percentage of Training Data Used curve is smoothed using a Gaussian kernel with a smoothness parameter of 0.8. (**b**) The Testing Accuracy vs. Percentage of Training Data Used curve is smoothed using a Gaussian kernel with a smoothness parameter of 0.5. (**c**) The Training Accuracy vs. Epochs curve, with 100% of Training Data Used, highlights our method’s faster convergence speed. (**d**) The Training Loss vs. Epochs curve, with 100% of Training Data Used, demonstrates that our method achieves lower training loss.

In classification tasks, the accuracy of both training and testing phases holds paramount importance in assessing the performance and generalization capability of classifier models. The training accuracy reflects how well the model learns from the provided data during the training phase, while the testing accuracy indicates how accurately the model can classify unseen data. In general, larger datasets tend to enhance model performance, assuming they encompass diverse and representative samples of the target population. However, the expansion of the dataset size can sometimes introduce a broader range of data quality issues. If left unaddressed, these issues may adversely affect the training accuracy. In the present study, we employed a ViT model pre-trained on the ImageNet-1K dataset [81], which consists of 1,281,167 training images. To emphasize the substantial difference in data size between our dataset, comprising only 1481 training images, and the ImageNet-1K dataset, this paper’s dataset is with a “limited” size. In contrast, the term “100% training data” is used to denote the utilization of the entire available dataset during the model training process. To demonstrate the stability of the model when working with a limited dataset, we conducted an experiment by randomly selecting subsets of the data with varying proportions of the original data size. The detailed data split is presented in Table A3. As captured in Table 3 or Figure 4a, regardless of the amount of training data utilized, the proposed model in this paper always has the higher training accuracy than the other state-of-art models.

By evaluating the model’s performance across these different data subsets, we aimed to assess its robustness and ability to maintain consistent results even when trained on reduced amounts of data. As illustrated in Figure 4a, we observed a decreasing trend in training accuracy with the enlargement of the training dataset. One potential remedy involves increasing the number of training epochs to accommodate the augmented data volume. Actually, upon closer examination of specific training data sizes, such as 50%, our proposed model demonstrates greater robustness compared to other models, exhibiting only minor differences in accuracy despite having the same increase in the amount of training images.

Testing accuracy is paramount in assessing the efficacy and reliability of a classification model, where a higher testing accuracy underscores the model’s capability to precisely classify unseen instances, thereby affirming its predictive prowess. As illustrated in Figure 4b, our model showcases its adeptness in identifying cervix types across diverse training data volumes, with testing accuracy improving in tandem with the expansion of the training dataset. The minimal data size required for accurate prediction using ViT models is influenced by various factors, such as task complexity, dataset diversity, and the specific architecture and hyperparameters of the employed ViT model. While there is no universally accepted threshold for the minimal data size, it is generally recommended to utilize thousands to tens of thousands of training examples to achieve satisfactory performance with ViT models. Notably, we observed more stable performance when employing more than 70% of the available data as illustrated in Figure 4b, with around 1000 images. This finding suggests that, for the cervical colposcopy dataset used in this study and the proposed model architecture, setting the minimal data size threshold at 70% is likely to yield satisfactory results.

The velocity at which deep learning neural networks train is subject to variation, influenced by factors including model intricacy, hardware performance, and the implementation of algorithmic enhancements such as adjustments to batch size and learning rate. Our model is distinguished by its comparatively minimal trainable parameters relative to other models evaluated. This is evident in Figure 4c, where our approach reaches peak training accuracy in fewer epochs, thereby expediting model convergence. This efficiency allows for the effective use of early stopping strategies to fine-tune the model without over-fitting, with the detailed training time seen in Table A2.

Additionally, monitoring training loss is indispensable during model training. Training loss measures the discrepancy between the predicted outcomes by the model and the actual labels, serving as an indicator of the model’s learning progress. A decreasing trend in training loss signifies the model’s increasing accuracy in capturing underlying patterns of the data. Demonstrated by Figure 4d, our model records the most substantial reduction in training loss, highlighting its superior capacity to learn from training data effectively.

### 4.4. Other Related Performance Metrics

**Figure 5:**
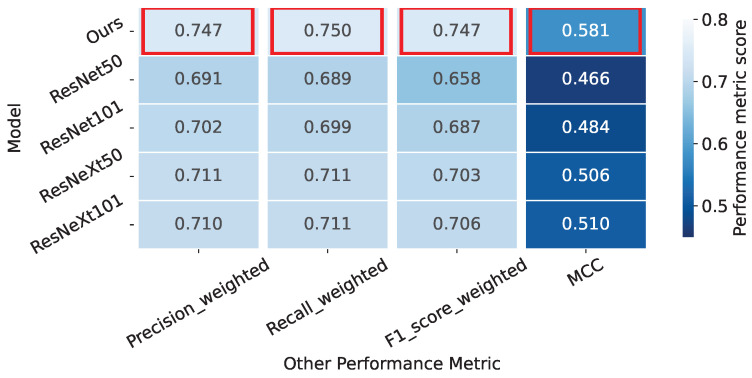
The performance metrics for each model with 100% of the training data. Each cell indicates the score of a specific performance metric for a given model. Models with the highest performance scores are outlined in red. The scores are computed individually for each cervix type and then averaged, weighted by the number of true instances for each type.

In medical imaging classification tasks, achieving high accuracy is crucial for precise diagnostic outcomes. Equally critical is the challenge presented by imbalanced datasets, characterized by a dominant prevalence of one class over others. To comprehensively evaluate a model’s performance in navigating such imbalanced datasets, it is important to consider evaluation metrics beyond mere accuracy, including precision, recall, F1 score, and MCC, ensuring a thorough assessment of model performance in handling imbalanced data. By showcasing various signature performance metrics in Figure 5, our method demonstrates superior performance across all proposed metrics.

### 4.5. Cross Validation

Cross validation is a crucial technique for evaluating the performance and generalization ability of deep learning models in cervical cancer detection using imaging classification [85,86,87,88]. By partitioning the dataset of cervical images into multiple subsets and iteratively training and testing the model on different combinations of these subsets, cross validation provides a more robust assessment of the model’s performance in identifying precancerous and cancerous lesions compared to a single train–test split. To test model reliability, we applied 5-fold cross validation on the complete dataset with 80% training data and 20% validation data, and our model achieved the best average performance metrics in accuracy, weighted precision, weighted recall, weighted F1 score, and Matthews Correlation Coefficient among the conventional models tested Table 4. These results demonstrate the robustness of our model and its ability to consistently outperform other approaches, validating its reliability in detecting cervical abnormalities across diverse subsets of the data.

## 5. Discussion

State-of-the-art models for cervical type classification have proven to be effective, with deep learning models reaching accuracy levels comparable to those of junior and even senior colposcopists in specific classification tasks [64]. Although these models have shown considerable success in terms of training accuracy, a focus solely on this metric without adequate testing accuracy or performance evaluation—particularly in scenarios involving limited datasets—may not fully capture the model’s efficacy. Moreover, as models grow in complexity with more layers and trainable parameters, the task of training them becomes increasingly demanding, especially in the context of limited available data for precise classification.

Pre-trained models often require fine-tuning to achieve accurate predictions on specific tasks. In our study, we investigated the performance of various non-pre-trained models in this paper, and compared them to the LoRA-ViT architecture. The results, as depicted in Figure 4, demonstrate that when properly tuned, pre-trained models exhibit superior performance compared to conventional CNN models. Importantly, our architecture not only highlights the advantages of pre-trained models but also emphasizes an efficient approach to fine-tuning them. Without appropriate fine-tuning, pre-trained models may yield similar or even inferior performance compared to non-pre-trained CNN models, potentially due to factors such as adversarial robustness [89]. By optimizing the fine-tuning process, our architecture accelerates training and enhances the accuracy of pre-trained models in typical scenarios. This finding underscores the importance of proper fine-tuning techniques when adapting pre-trained models to specific tasks, as it can significantly improve their performance and efficiency.

Our method overcomes existing challenges by achieving unparalleled accuracy on test datasets that have not been seen during training, regardless of the amount of data used for training. Furthermore, it surpasses current models, particularly in the demanding task of Type 1 cervical classification, through the incorporation of a robust evaluation framework that ensures both reliability and effectiveness in its performance assessment. Also, our method enables the efficient training of the transformer layer without necessitating extensive computational resources. For various downstream tasks, training low-rank matrices with a reduced parameter count is sufficient, facilitating the use of pre-trained weights across different tasks and thereby simplifying the training process. This strategy significantly speeds up the training period, obviating the need for gradients of pre-trained weights and their optimizer states, which in turn, boosts training efficiency and reduces the demand on hardware. Furthermore, the integration of trained low-rank matrices with pre-trained weights merges multi-branch architectures into a singular streamlined branch, effectively eliminating inference latency. Despite comprising only 0.05% of the trainable parameters of the original ViT, our lightweight model demonstrates its capability to efficiently manage larger datasets and conduct neural network training for medical imaging tasks with exceptional efficiency. And with cross validation, it helps to mitigate over-fitting, which is particularly important in medical imaging tasks, where the available datasets may be limited, enables the selection of optimal hyperparameters that maximize the model’s accuracy and sensitivity in detecting cervical abnormalities, and ensures that the models are robust and generalizable to diverse patient populations and imaging conditions encountered in real-world clinical settings.

Moving forward, our approach maintains compatibility with parameter-efficient fine-tuning methodologies, including Adapters [90,91], Prefix-Tuning [92,93], and Visual Prompt-Tuning [94,95], supported by dimension reduction techniques, such as some previous application of Uniform Manifold Approximation and Projection [96,97,98,99,100] in clustering, and auto-encoder and variational auto-encoder models. These strategies are designed to fine-tune a restricted subset of parameters, introduced in a gradual manner, thereby eliminating the necessity to adjust all parameters within a pre-trained model and capture the most salient features of the data while reducing noise and computational complexity. The harmonious integration with these techniques substantially refines the tuning process. It achieves this by diminishing the requirements for computational and storage resources, all while ensuring there is no disruption to the existing framework. Also, we aim to enhance Vision Transformer adapters to facilitate multi-task learning by encapsulating task-specific knowledge and relationships. This will allow the adapters to be generalized and applied to novel tasks and domains without requiring extensive retraining or fine-tuning, thereby improving the efficiency and flexibility of the model in handling diverse computer vision challenges [101,102].

However, several critical areas demand our focus moving forward. A paramount concern lies with the dataset issue, highlighting the necessity for not only larger but also higher-quality datasets. It is notable that within all models’ classification outcomes, the accuracy for Type 1 significantly lags behind that of Types 2 and 3, suggesting potential quality issues with the Type 1 data. With higher-quality Type 1 data and labels, the results are bound to significantly improve. Furthermore, investigating larger model architectures could lead to significant enhancements in model generalizability, potentially increasing accuracy even with limited training datasets. However, such advancements would require more substantial computational resources. Pursuing these avenues is essential for improving the accuracy and reliability of medical imaging classification systems. To support the training of these more sophisticated models, there is also a pressing need for better hardware, particularly more powerful GPUs. Advances in hardware technology will not only expedite the training process but also enable the handling of more complex models and larger datasets with greater ease. These enhancements are vital for pushing the boundaries of what is currently achievable in medical imaging classification, paving the way for more accurate, efficient, and reliable diagnostic tools in the future.

## Figures and Tables

**Table 1:** A breakdown table of the MobileODT dataset.

Dataset	Type 1	Type 2	Type 3
Training set ^1^	250	781	450
Testing set ^2^	87	265	160

This set is optimized to speed up the training process ^1^; Hold out data ^2^.

**Table 2:** Training parameters comparison.

Model	Number of Trainable Parameters	Model	Number of Trainable Parameters
Ours	**0.15 M**	AlexNet	60.0 M
ResNeXt-50	25.0 M	ViT-base	82.0 M
ResNet-50	25.6 M	ResNeXt-101	88.8 M
DenseNet	33.0 M	VGG-19	144 M
ResNet-101	44.6 M	ViT-huge	289.5 M

All the models listed above: Batch size = 32, Learning Rate by scheduler = CosineAnnealingLR (optimizer, cfg.epochs, 3 × 10^−6^) with initial rate as 1 × 10^−3^.

**Table 3:** Best accuracy and training loss of all experimental models with 100% training data used.

Model	Training Acc.	Testing Acc.	Training Loss
AlexNext	53.9%	52.5%	N/A
VGG-19	53.0%	52.9%	N/A
GooGleNet	96.8%	72.3%	N/A
DenseNet-121	97.3%	72.5%	N/A
ResNet-50	94.5%	68.9%	1.15
ResNet-101	96.0%	69.9%	1.11
ResNeXt-50	95.6%	71.1%	1.13
ResNeXt-101	94.9%	71.3%	1.14
Ours	**98.9%**	**75.0%**	**0.31**

**Table 4:** Testing data performance metric with 5-fold cross validation.

Model	Accuracy	Precision ^1^	Recall ^1^	F1-Score ^1^	MCC
ResNet-50	0.682 ± **0.006**	0.686 ± 0.010	0.682 ± **0.006**	0.666 ± 0.011	0.454 ± **0.011**
ResNet-101	0.686 ± 0.012	0.688 ± 0.014	0.682 ± 0.012	0.672 ± 0.016	0.461 ± 0.023
ResNeXt-50	0.694 ± 0.007	0.690 ± **0.007**	0.695 ± 0.007	0.685 ± **0.009**	0.481 ± 0.015
ResNeXt-101	0.695 ± 0.013	0.705 ± 0.020	0.695 ± 0.013	0.681 ± 0.017	0.480 ± 0.022
**Ours**	**0.734** ± 0.011	**0.734** ± 0.012	**0.734** ± 0.011	**0.724** ± 0.016	**0.549** ± 0.020

Weighted metric ^1^; Each cell stands for (average metric ± standard deviation).

**Figure A1:**
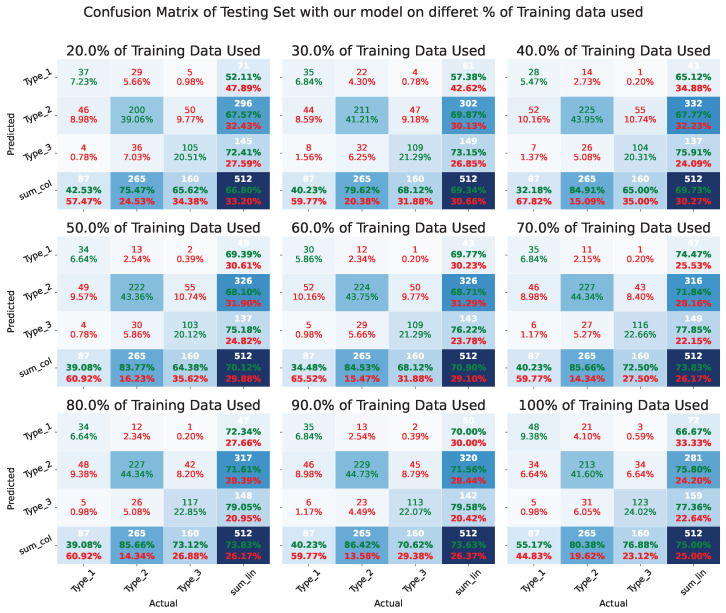
Confusion matrix of our method with different % of training data used.

**Figure A2:**
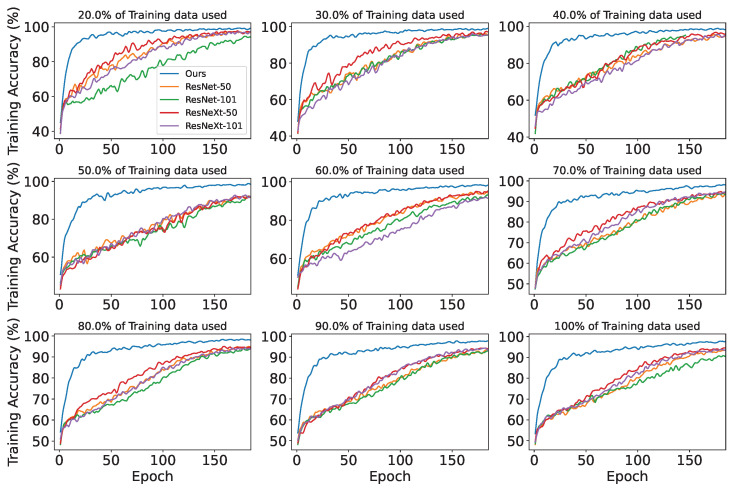
Training accuracy vs. epoch.

**Figure A3:**
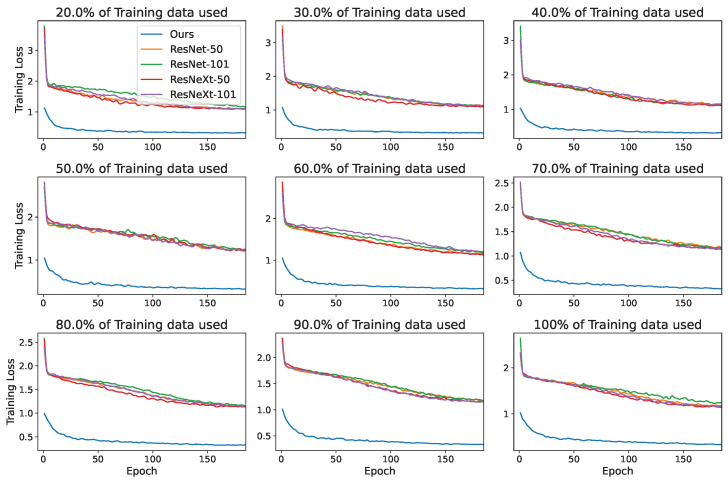
Training loss vs. epoch.

**Figure A4:**
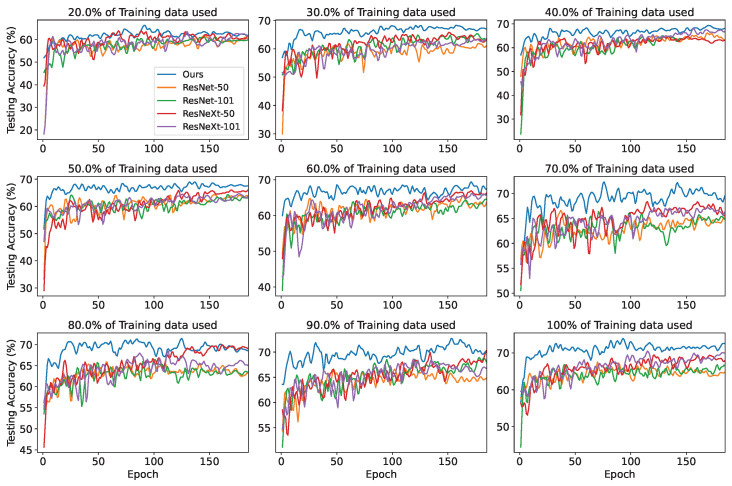
Testing accuracy vs. epoch.

**Figure A5:**
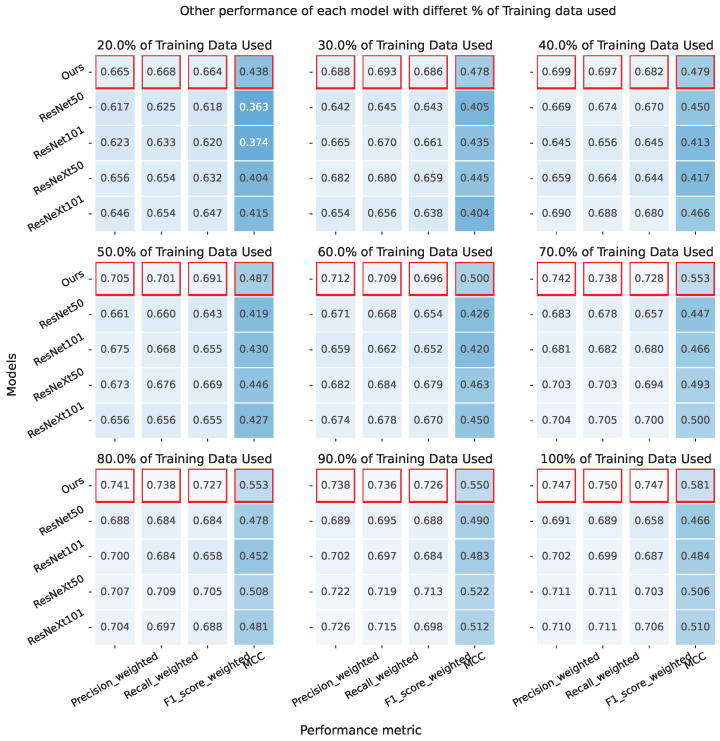
Other model performance metric with % training data used. Models with the highest performance scores are outlined in red. Color bar legend seen in Figure 5.

## Data Availability

Raw data are in Intel & MobileODT Cervical Cancer Screening competition as of 21 February 2024. Models and code used in our analysis are available in the paper’s GitHub repository: https://github.com/Deep-Fusion-Innovators/paper-Cervical-Cancer-Detection. This repository also contains Jupyter notebooks that can be run to reproduce the results presented here. Pipeline is coded in Python3/C++. ViT-large model used in this study is pre-trained on LAION-2B image-text pairs using OpenCLIP.

## Abbreviations

The following abbreviations are used in this manuscript:

LoRA	Low-Rank Adaptation
ViT	Vision Transformer
MCC	Matthew Correlation Coefficient
ResNet	Residual Network
Pap	Papanicolaou
HPV	Human Papillomavirus Infection
CNN	Convolutional Neural Network
GPU	Graphics Processing Unit
ReLU	Rectified Linear Unit
CAD	Computer-Aided Diagnosis
TP	True Positive
TN	True Negative
FN	False Negative
FP	False Positive

## Appendix A. Additional Tables

**Table A1:** A summary table of the related works on the cervix colposcopy image database.

Reference	Dataset	Methods	Metrics
Mustafa and Dauda [63]	Undisclosed dataset	CNN-Adam	Accuracy = 0.90 ^1^, 0.74 ^2^
		CNN-SGD	Accuracy = 0.83 ^1^
		CNN-RMSProp	Accuracy = 0.88 ^1^
Liu et al. [64]	Undisclosed data	Clinic-based model	Accuracy = 0.68 ^3^
			AUC = 0.7 ^3^
			Specificity = 0.71 ^3^
		ResNet-50	Accuracy = 0.8 ^3^
			AUC = 0.95 ^3^
			Accuracy = 0.88 ^3^
			Specificity = 0.87 ^3^
Peng et al. [65]	Undisclosed data	ResNet-50	Accuracy = 0.80 ^1^
			Specificity = 0.78 ^1^
		DenseNet121	Accuracy = 0.76 ^1^
			Specificity = 0.77 ^1^
		VGG16	Accuracy = 0.86 ^1^
			Specificity = 0.90 ^1^
Cruz et al. [103]	MobileODT	CXNN	Accuracy = 0.77 ^1^
			Accuracy = 0.664 ^2^
Gorantla et al. [88]	MobileODT	CervixNet	Accuracy = 0.97 ^1^
			Specificity = 0.98 ^1^
			Precision = 0.98 ^1^
			F1-score = 0.97 ^1^
Aina et al. [104]	MobileODT	AlexNet	Accuracy = 0.63 ^2^
		SqueezeNet	Accuracy = 0.63 ^2^
Payette et al. [105]	MobileODT	Residual CNN-32L ^4^	Accuracy = 0.58 ^2^
		Residual CNN-53L ^4^	Accuracy = 0.55 ^2^
		CNN-32L-Drop ^4^	Accuracy = 0.56 ^2^
		CNN-55L-Drop ^4^	Accuracy = 0.50 ^2^
Darwish et al. [84]	MobileODT	ViT	Accuracy = 0.91 ^1^
		ViT-SPT/LSA	Accuracy = 0.91 ^1^
			Accuracy = 0.38 ^3^

Training set ^1^; Validation set ^2^; Testing set ^3^; L = Layer ^4^.

**Table A2:** Training time comparison with entire dataset.

Model	Training Time (mins/10 epochs)	Convergence Epochs	Converged Training Time (h)
Ours	33.8	**25**	**14.0**
ViT-Large	56.3	96	90.08
ResNet-50	33.7	185	103.9
ResNet-101	**30.3**	210	106.1
ResNeXt-50	32.1	162	86.67
ResNeXt-101	31.9	149	79.2

**Table A3:** A breakdown table of training data size.

% of Training Data	Type 1	Type 2	Type 3
20%	50	156	90
30%	75	234	135
40%	100	312	180
50%	125	390	225
60%	150	468	270
70%	175	546	315
80%	200	624	360
90%	225	702	405
100%	250	781	450
